# Fusion Cell Markers in Circulating Tumor Cells from Patients with High-Grade Ovarian Serous Carcinoma

**DOI:** 10.3390/ijms232314687

**Published:** 2022-11-24

**Authors:** Anna Paula Carreta Ruano, Andrea Paiva Gadelha Guimarães, Alexcia C. Braun, Bianca C. T. C. P. Flores, Milena Shizue Tariki, Emne A. Abdallah, Jacqueline Aparecida Torres, Diana Noronha Nunes, Bruna Tirapelli, Vladmir C. Cordeiro de Lima, Marcello Ferretti Fanelli, Pierre-Emmanuel Colombo, Alexandre André Balieiro Anastácio da Costa, Catherine Alix-Panabières, Ludmilla Thomé Domingos Chinen

**Affiliations:** 1 International Research Center, A.C.Camargo Cancer Center, São Paulo 01508-010, Brazil; 2 Medical Oncology Department, A.C.Camargo Cancer Center, São Paulo 01525-010, Brazil; 3 Núcleo de Pesquisa e Ensino da Rede São Camilo, São Paulo 04014-002, Brazil; 4 Department of Surgical Oncology, Institut du Cancer de Montpellier, ICM-Val d’Aurelle, 34090 Montpellier, France; 5 Laboratory of Rare Human Circulating Cells (LCCRH), Department University Medical Centre of Montpellier, 34295 Montpellier, France; 6 CREEC/CANECEV, MIVEGEC (CREES), University of Montpellier, CNRS, IRD, 34000 Montpellier, France

**Keywords:** circulating tumor cells, cell fusion, hybrid cells, CD45, ovarian cancer, in situ hybridization

## Abstract

Cancer is primarily a disease in which late diagnosis is linked to poor prognosis, and unfortunately, detection and management are still challenging. Circulating tumor cells (CTCs) are a potential resource to address this disease. Cell fusion, an event discovered recently in CTCs expressing carcinoma and leukocyte markers, occurs when ≥2 cells become a single entity (hybrid cell) after the merging of their plasma membranes. Cell fusion is still poorly understood despite continuous evaluations in in vitro/in vivo studies. Blood samples from 14 patients with high-grade serous ovarian cancer (A.C. Camargo Cancer Center, São Paulo, Brazil) were collected with the aim to analyze the CTCs/hybrid cells and their correlation to clinical outcome. The EDTA collected blood (6 mL) from patients was used to isolate/identify CTCs/hybrid cells by ISET. We used markers with possible correlation with the phenomenon of cell fusion, such as MC1-R, EpCAM and CD45, as well as CEN8 expression by CISH analysis. Samples were collected at three timepoints: baseline, after one month (first follow-up) and after three months (second follow-up) of treatment with olaparib (total sample = 38). Fourteen patients were included and in baseline and first follow-up all patients showed at least one CTC. We found expression of MC1-R, EpCAM and CD45 in cells (hybrid) in at least one of the collection moments. Membrane staining with CD45 was found in CTCs from the other cohort, from the other center, evaluated by the CellSearch^®^ system. The presence of circulating tumor microemboli (CTM) in the first follow-up was associated with a poor recurrence-free survival (RFS) (5.2 vs. 12.2 months; *p* = 0.005). The MC1-R expression in CTM in the first and second follow-ups was associated with a shorter RFS (*p* = 0.005). CEN8 expression in CTCs was also related to shorter RFS (*p* = 0.035). Our study identified a high prevalence of CTCs in ovarian cancer patients, as well as hybrid cells. Both cell subtypes demonstrate utility in prognosis and in the assessment of response to treatment. In addition, the expression of MC1-R and EpCAM in hybrid cells brings new perspectives as a possible marker for this phenomenon in ovarian cancer.

## 1. Introduction

Globally, cancer is the leading cause of death [1]. In 112 of 183 countries, it is the number-one cause of death before age 70, and in at least 23 countries, it ranks third or fourth, according to World Health Organization (WHO) estimates [2]. Ovarian cancer is one of the deadliest gynecological cancers: in 2020, WHO estimated that it was responsible for 313,959 new cases and 207,252 deaths [2]. In Brazil in 2020, ovarian cancer accounted for 6650 new cases and 3921 deaths [3].

Precision medicine based on the evaluation of primary tumor characteristics is complicated, as it must account for intra-tumor heterogeneity and tumor changes over time [4]. Circulating tumor cells (CTCs), one of the circulating biomarkers of the “liquid biopsy” [5], have been studied since 2004. The U.S. Food and Drug Administration (FDA) has approved the CellSearch^®^ system for analysis of CTCs in metastatic breast, colorectal and prostate cancers. This system involves isolation by enrichment with epithelial cell-specific antibodies (EpCAM), and CD45 for leukocyte depletion [6,7,8]. However, the studies conducted since then have proven CTCs to be highly heterogeneous and difficult to isolate solely by EpCAM and CD45. Many studies have shown that CTCs that undergo epithelial–mesenchymal transition can express epithelial, mesenchymal and stem cell markers [9,10]. Therefore, methods based on physical and morphological properties of CTCs may be more reliable. In addition, by using an epithelial marker-independent enrichment technique, it may be possible to identify CTC clusters or circulating tumor microemboli (CTM) composed of only CTCs or CTCs escorted by immune cells. CTM is more likely to survive in the bloodstream, to overcome cytotoxic treatment and to form metastases than CTCs [11]. Thus, epithelial marker-independent enrichment methods for CTCs and CTM can offer new opportunities to study their heterogeneity. Techniques based on their physical characteristics, such as size, density, deformability and electrical properties, will offer additional insight [12,13].

Cell fusion in CTCs is defined as the expression of both carcinoma and leukocyte markers [14]. It is a feature that can be evaluated by marker-independent enrichment methods. In 1911, Professor Otto Aichel was the first to propose that cancer cells can fuse themselves with white blood cells, spread through the peripheral circulation and initiate the metastatic process [15]. Conceptually, cell fusion occurs when two or more cells become a single entity after the merging of their plasma membranes [16,17]. The process is responsible for the formation of new organisms via fertilization or mating, as well as for the formation of fused cells such as hybridomas (myeloma cells fused with lymphocytes) [17]. It is also employed in the production of monoclonal antibodies.

Despite the continuous evaluation of cell fusions by many in vitro and in vivo studies [18,19], this multistep process involving cell–cell recognition, cell adhesion and membrane fusion [20] is still poorly understood. Cell fusion is an important and highly controlled process, key to embryonic development and maintaining homeostasis. It has also gained attention for its proposed role in cancer progression, as a cooperative mechanism underlying metastasis and drug resistance [21]. The process allows cancer cells to fuse with diverse non-tumor cells, including stromal and epithelial cells and macrophages [22,23].

Melanocortin receptor 1 (MC1-R) is a five-transmembrane G-protein-coupled receptor that allows for the influx of extracellular calcium and subsequent activation of inositol triphosphate. MC1-R has myriad ligand affinities and downstream effects and can be found in many cell types, including melanoma, epithelial and endothelial and immune cells [24,25] Some authors have reported fusion events between macrophages and melanoma cells [26,27].

Polyploidy is another signal of cell fusion. Polyploid cells contain two or more sets of homologous chromosomes. Polyploidy is a spontaneous event that contributes to normal organogenesis, tissue repair and tissue differentiation [28,29]. It can contribute to the formation of centrosomal aberrations, which leads to aneuploid genomes [30]. As a result, fusion hybrids can provide a survival advantage, making it easier for tumor cells to be cloaked with innate epitopes and evade the immune system, facilitating survival in the bloodstream and parenchymal infiltration [23].

In the present study, we observed fused cells using two different CTC identification systems. The first was ISET^®^, which isolates CTCs by size through filtration and cytopathological analysis. The second was the CellSearch system^®^, which identifies CTCs of epithelial origin using magnetic beads.

Here we report a series of 14 patients with high-grade serous ovarian cancer. The primary outcome was to determine the presence of cell fusion (hybrid cells) in CTCs. This was performed using immunocytochemical analysis to detect the expression of CD45 alone or with MC1-R [24,25,31] or EpCAM, a marker commonly used to isolate CTCs from epithelial tumors [32]. The secondary outcomes were to test the association of fusion markers in CTCs with recurrence-free survival (RFS) and overall survival (OS). We also evaluated the presence of centromere polyploidy of chromosome 8 at baseline to confirm the presence of hybrid cells and tested its association with RFS and OS. Finally, we reported three cases of ovarian cancer from another medical center that were evaluated by the CellSearch system^®^ to confirm our findings of hybrid cells using another method.

## 2. Results

### 2.1. Clinical-Pathological Data

Fourteen patients were included, all of whom had high-grade serous ovarian cancer and were undergoing platinum-based chemotherapy with maintenance PARP inhibitor treatment (olaparib). The median age was 45 years (range 29–55). All patients were treated with carboplatin and paclitaxel, 64.3% of patients with six cycles and 14.3% with eight cycles. All patients included in the study had already relapsed once before being included in the study and the most frequent locations for relapse were pelvic (28.6%), pelvic and mediastinum (14.3%), mediastinum (7.1%), pelvic/retroperitoneum (7.1%) and retroperitoneum (7.1%). Before baseline CTC collection, eight patients (57.1%) had optimal cytoreduction and three (21.4%) had suboptimal cytoreduction. There were no cytoreduction data for the remaining patients. Eight patients (57.1%) had a BRCA1 or BRCA2 mutation, three (21.4%) were BRCA1 and BRCA2 wild type, and three had not been tested. The CA125 tumor marker commonly used to indicate the presence of ovarian tumor had a median of 268 and a mean of 1141.57 (78-6056; SD = 2184; laboratory reference value = 35U/mL). Six of fourteen patients had disease relapse before the first follow-up. Four patients had disease relapse in between the first and second follow-up. After the second follow-up, only one patient relapsed (Table 1).

### 2.2. Analysis of Protein Expression

The median number of CTCs detected by ISET at baseline was 0.58 CTCs/mL (range 0.33–9.91). At the first follow-up, the median was 0.60 CTCs/mL (range 0.20–7.20), and at the second follow-up, the median was 1 CTCs/mL (range 1.00–8.58). Only 10 patients had blood collected at the second follow-up: 1 dropped out of the study due to disease recurrence in the first collection period and the other 3 were lost to follow-up.

**Figure 1:**
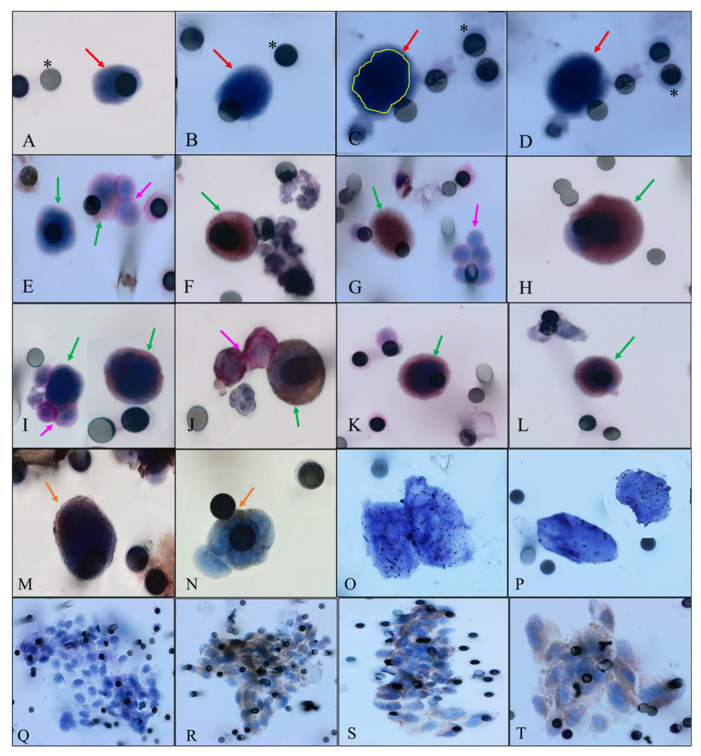
Representative images. Green arrows indicate hybrid cells, pink arrows represent leukocytes, red arrows represent CTCs, orange arrows indicate hybrids cells stained with CD45, yellow circle indicate cell nucleus and asterisks represent membrane pore. (**A**,**B**) CTCs without any staining, visualized with hematoxilin. (**C**,**D**)The same CTC without any staining, in (**C**), nuclei defined by yellow line. (**E**) Hybrid cells. One positive for MC1-R (brown membrane) and one in a microemboli, stained with CD45 with two leukocytes (stained with CD45, visualized by permanent red). (**F**) Hybrid cell positive for MC1-R (brown membrane). (**G**) Hybrid cell positive for EpCAM (brown membrane) and a cluster of leukocytes (stained with CD45, visualized by permanent red). (**H**) Hybrid cell double positive for EpCAM (brown membrane) and MC1-R (visualized by permanent red). (**I**) Two hybrid cells. One visualized alone double stained with EpCAM and CD45 (brown and red) and the other surrounded by three leukocytes stained with CD45 (visualized by permanent red). (**J**) Hybrid cell stained with MC1-R, visualized by DAB and two leukocytes (stained with CD45, visualized by permanent red). (**K**,**L**) Hybrid cells positive for MC1-R/CD45 (brown and red membrane). (**M**,**N**) Hybrids cells stained with CD45 in just one side of the cytoplasmatic membrane, visualized by DAB. (**O**,**P**) CTCs positive for CEN8, indicating the presence of polyploidy. (**Q**) Microemboli visualized with hematoxylin. (**R**–**T**) Microemboli from patient positive for MC1-R (brown membrane). Images were taken at ×400 and ×600 magnification using a light microscope (Research System Microscope BX61—Olympus, Tokyo, Ja-pan) coupled to a digital camera (SC100—Olympus, Tokyo, Japan).

As stated in materials and methods, a cell was considered hybrid if stained positive for CD45 with or without EpCAM or MC1-R. At baseline, 4/14 patients were positive for CD45, 3 were also positive for MC1-R and 1 for EpCAM. At the first follow-up, CD45 was found in 6/14 patients, none expressed EpCAM, and all CD45 positive cells were also positive for MC1-R. In the second follow-up, CD45 was positive in 2/10 cells and both cells were positive for MC1-R. One of these cells was positive (in another spot analyzed) for EpCAM (Figure 1).

Interestingly, MC1-R was found in cells that did not stain for CD45. At baseline, in 14 patients evaluated, 10 expressed MC1-R, although only 3 were positive for CD45. At first follow-up, 10/14 cells were positive and in the second, 5/10 were MC1-R positive. Patients who presented CTCs/mL above the median more frequently expressed MC1-R (*p* = 0.07 at baseline; *p* = 0.085 at first follow-up; *p* = 0.008 at second follow-up). Interestingly, CTCs counts in the three moments were not associated with CA125 above the median. For more information concerning clinical data and protein expression in CTCs, please see Supplementary Material, Table S1.

### 2.3. CEN8

The expression of chromosome 8 (CEN 8) to assess the presence of polyploidy cell fusion (hybrid cell) was tested in all patients only at baseline. Of the 14 patients, 9 had CTCs that were positive for CEN8 expression (64.3%) and 2 patients had CTCs negative for CEN8 (14.28%). We evaluated only one spot on the ISET membrane of each patient for the CEN8 analysis. Samples from 3 patients had no cells in the evaluated spot. It may be that if we had evaluated more spots, we would have found positivity in the 3 patients whose spots had no cells. The positivity rate for this probe was 81.8% (9/11).

### 2.4. CTCs and Recurrence-Free Survival (RFS)

As described in the methodology, we considered that a CTC showed cell fusion when it expressed CD45 alone or co-expressed with MC1-R, which is a marker used in the literature to describe cell fusion [24,25], or CD45 co-expressed with EpCAM, which is an epithelial cell marker [33]. However, we evaluated each protein (CD45, MC1R and EpCAM) independently to test its impact in RFS and OS.

Median RFS time was 8.13 months (0.625 vs. 32.86 months). CTC count was not associated with RFS. Patients with CTC/mL counts above the median (0.58 CTCs/mL; range: 0.33–9.91 CTCs/mL) at baseline had a higher number of relapse sites (*p* = 0.025). There was no correlation of any other variable with the number of sites of relapse.

**Figure 2:**
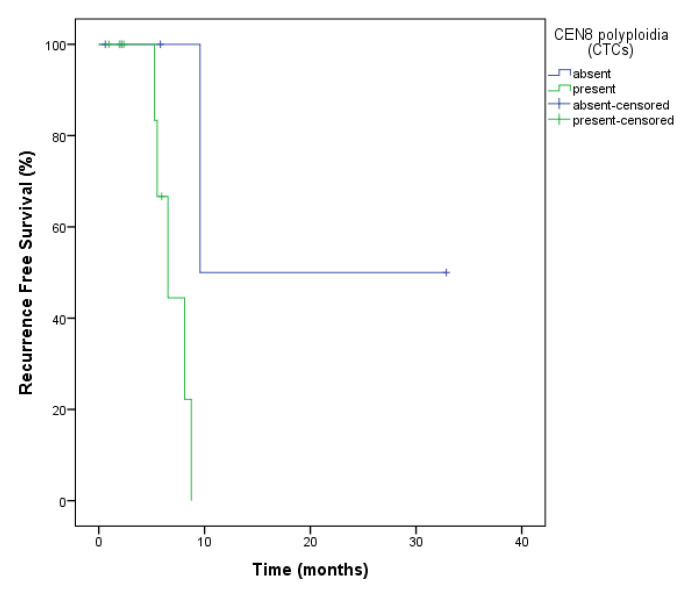
Recurrence-free survival analysis of ovarian cancer patients for CEN8 expression in CTCs (hybrid cells) at the baseline. The presence of polyploidy in the CEN8 showed a worse RFS (7.0 months vs. 21.21 months, *p* = 0.035) in relation to those who did not.

Patients whose CTCs presented polyploidy as evaluated by CEN8 analysis (hybrid cell) had a worse RFS (7.0 months vs. 21.21 months, *p* = 0.035) in relation to those who did not (Figure 2).

Notably, although CD45 was used as our fusion marker, as all proteins were evaluated together with it, CD45 expression alone was not associated with survival. On the other hand, MC1-R was associated with RFS, suggesting MC1-R as a poor prognosis protein in ovarian cancer.

**Figure 3:**
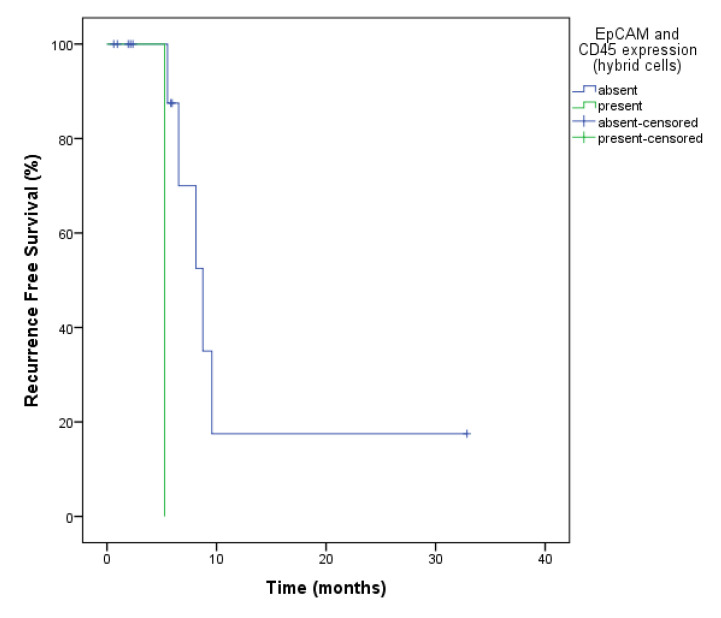
Recurrence-free survival analysis of ovarian cancer patients for EpCAM and CD45 expression in hybrid cells at the baseline and at second follow-up. The expression of this markers showed a worse RFS of 5.26 months vs. 12.2 (*p* = 0.005) in relation to those who did not.

At baseline, patients who expressed MC1-R had a median RFS of 14.07 vs. 6.8 compared to those who did not (*p* = 0.30). For EpCAM, the only patient who expressed this protein at baseline and at second follow-up, always together with CD45, which makes the cell doubtless hybrid, had a RFS of 5.26 months vs. 12.21 for those who did not express it, at two timepoints (baseline and second follow-up) (*p* = 0.005) (Figure 3).

**Figure 4:**
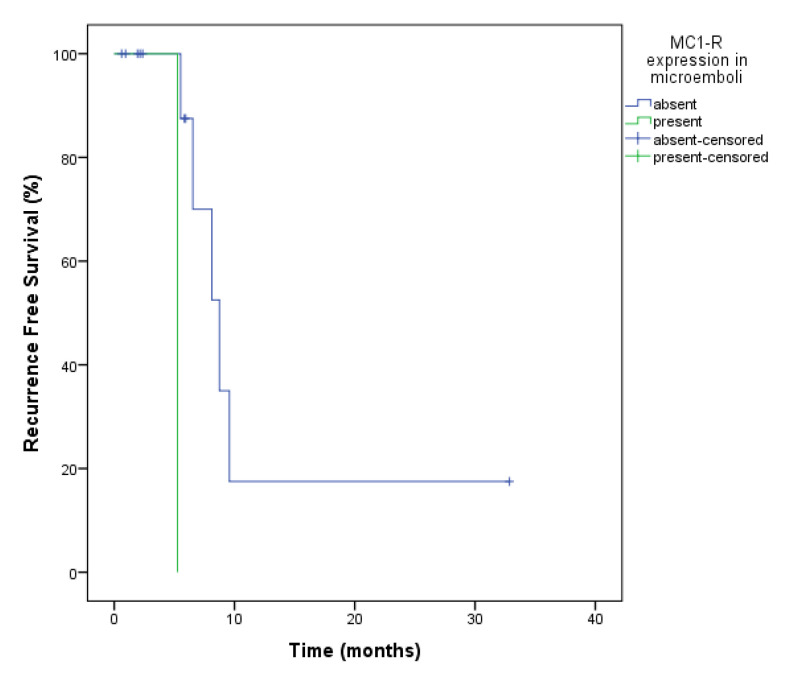
Recurrence-Free Survival analysis of ovarian cancer patients for MC1-R expression in CTM. The presence of a microemboli with MC1-R expression at the second follow-up was also determinant for worse RFS (5.26 vs. 13.1 months; *p* = 0.008).

The presence of a microemboli in the first follow-up and second follow-ups were associated with a shorter RFS (5.26 vs. 13.1 months; *p* = 0.008). Curiously, the only protein found in microemboli was MC1-R. Expression of MC1-R in the microemboli in the first and second follow-ups was associated with RFS. At this both moments, patients that did not express MC1-R had a median RFS of 13.1 months, while those who expressed had median RFS of 5.26 months (*p* = 0.008) (Figure 4).

### 2.5. CTCs and Overall Survival

Median OS was 20.4 months (0.95–35.26 months). Patients who had CTCs expressing MCR-1 had an OS of 30.31 months compared to 13.37 months for those who did not have it (*p* = 0.059). CD45 expression was not associated with OS, 20.42 months vs. 24.11 months (*p* = 0.91).

Biomarkers evaluated in samples from the first follow-up were not associated with OS. In the second follow-up, patients with MCR-1 expression presented a median OS of 20.42 months vs. 27.41 months for those who did not express it (*p* = 0.7).

### 2.6. CTCs Detected Using the CellSearch^®^

**Figure 5:**
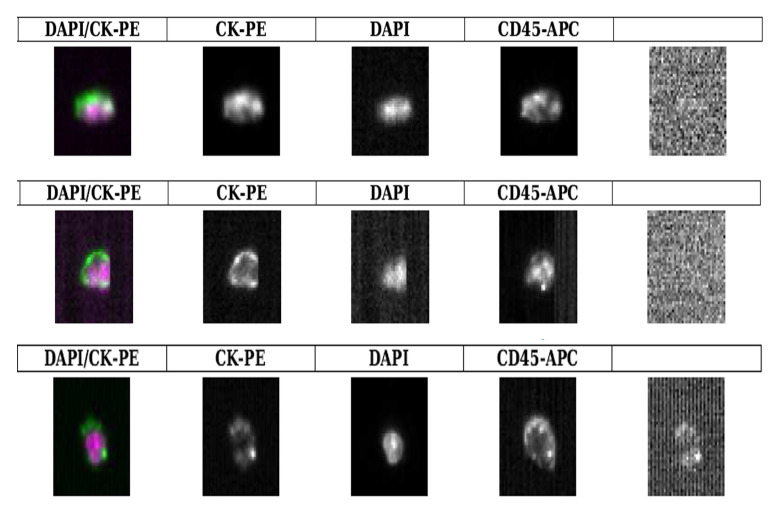
CTCs isolated from 3 different patients with locally advanced ovarian cancer with peritoneal carcinomatosis but no distant metastases (M_0_). These photos are representative of dual stained CTCs detected using the CellSearch^®^ system: dual CK^(+)^ CD45^(+)^ nucleated cells after EpCAM-based enrichment step.

Samples from three patients with ovarian cancer attended at the Department of Surgical Oncology, Institut du cancer de Montpellier, ICM-Val d’Aurelle, Paris, France, were analyzed in parallel with the A.C Camargo Cancer Center, São Paulo, Brazil, cohort to confirm what we found with the size-based technology “ISET”. We asked researchers to look at the cells that normally are discarded by the gold standard CellSearch^®^ system, due to CD45 expression. Surprisingly, some cells had both: CD45 and epithelial markers, as well as morphological features of CTCs, confirming that fusion phenomenon is observed independently of the method used to detect CTCs (Figure 5).

## 3. Discussion

In the present study we could observe fused cells using two different CTC identification systems. The first is ISET^®^, which isolates the CTCs by size through filtration and cytopathological analysis. Circulating tumor cells were defined as cells presenting all the following criteria: (i) irregularity of the nuclear contour; (ii); presence of a visible cytoplasm; (iii) nuclear size equal or larger than two pores (equal or larger than 16 μm); and (iv) high nuclear-to-cytoplasmic ratio (>0.8) [9]. Leukocytes were defined following the criteria: (i) cytoplasm with azurophilic and specific granules; (ii) measure from 9μm to 15 μm; positive staining for CD45 [34].

The second is the CellSearch system^®^, which identifies and enumerates CTCs of epithelial origin by magnetic beads labeled with EpCAM.

The high detection rate of CTCs in the present patient population and the correlation of baseline CTC counts with the number of relapse sites suggest that ISET can be used to identify, quantify and characterize CTCs and hybrid cells in patients with high grade serous ovarian cancer. Polyploidy was also observed in the isolated cells and correlated with poor RFS. MC1-R, a protein used to identify fusion events in melanoma, was found in CTCs and CTM and shows promise as a prognostic marker in ovarian cancer.

Our study shows that the ISET method can be used to identify not only CTCs but also hybrid cells, and that, according to other authors [35,36], it can be better than label-dependent methods to observe CTCs. Some studies have already demonstrated the effectiveness and specificity of cytopathological analysis of CTCs made after ISET isolation. One study with renal tumors showed that 104 of 125 CTCs identified by cytopathological analysis expressed the identical VHL mutation as detected in the primary tumor [35]. Another study, also from the same group, showed that the detection of CTCs from a prostate tumor, analyzed by cytopathology without any other molecular characterization, was capable of identifying patients at higher risk of recurrence after prostatectomy. According to this study, the cytopathology specificity was 100%, while the sensitivity was 72% [36].

Searles et al. hypothesized that cell fusion might cause chromosomal instability. They used a Cre-loxP system to observe the molecular exchange of information between cancerous and non-cancerous cells and postulated that cells that express Cre may induce loxP recombination in normal reporter cells. Surprisingly, all marked cells exhibited hyperploidy and resulted from a cell fusion event [37]. Thus, we decided to analyze the presence of chromosomal instability in CTCs, since ovarian cancer is characterized by copy number and structural variations [38]. Recent studies have shown that chromosome 8 triploid and tetraploid CTCs are resistant to intrinsic drugs in gastric cancer, nasopharyngeal carcinoma, and rectal cancer [39]. Chromosome 8 aneuploidy is common in several neoplastic diseases. According to a study by Zhang et al., CTC triploids could be detected in most patients with newly diagnosed nasopharyngeal carcinoma, but the number of polyploid cells was significantly higher in patients with recurrence and metastasis. They also postulated that the copy number of chromosome 8 was closely related to the effectiveness of chemotherapy and resistance to treatment [40]. Chromosome aneuploidy is known to occur in several tumor types, and many studies have shown that patients with aneuploid tumor cells had poorer outcomes [41]. Li et al. demonstrated that triploid CTCs were associated with intrinsic resistance to drugs, while tetraploid and multiploid CTCs were related to acquired resistance to paclitaxel or cisplatin in gastric cancer. Clinical studies conducted in patients with gastric cancer indicated that rare cells with trisomy 8 showed intrinsic resistance to cisplatin, while multiploid cells demonstrated acquired resistance [42]. Here, we showed that CEN8 was related to poor RFS in ovarian cancer (*p* = 0.035). Maybe, in this type of tumor, CEN8 can be a marker of fusion event, as it is believed that hybrid cells are related to poor outcomes, but further studies are necessary to prove this.

MC1-R is thought to play a role in melanoma progression through the activation of MET, a proto-oncogene and key regulator of metastasis in many cancers. Clawson et al., working with cultured, enriched CTCs from melanoma patients, observed large CTCs (generally 20–30 µm) in early-stage patients. The isolated CTCs showed aberrant expression of melanocytic differentiation markers, as well as pan-cytokeratin. Two subpopulations were observed based on immunofluorescence staining: one subpopulation of cells stained for pan-cytokeratin and CD45 (50%) (hybrid cells) and the other stained only for pan-cytokeratin (50%) [43]. Likewise, Itakura et al. described two subgroups of melanoma-related cells in sentinel lymph nodes, analogous to the two subpopulations described by Clawson: one composed of cells consistent with immature melanocytes and the other composed of cells consistent with immature melanocytes that also expressed leukocyte/macrophage markers (fusion event) [44].

Here, we describe for the first time the expression of MC1-R in CTCs from ovarian cancer patients and its correlation to poor RFS when expressed in CTM at the first and second follow-ups. Because MC1-R was observed in CTCs with CD45 expression, it may be useful as a marker of hybrid cells in ovarian cancer as it is in melanoma [45]. As was demonstrated in other tumor types [46,47,48], CTC clusters were also related to poor outcome in ovarian cancer.

The fusion phenomenon is not a novel discovery, but its finding in CTCs of patients with ovarian cancer is unique. To the best of our knowledge, this is the first study describing the cell fusion phenomenon observed under light microscopy, allowing the visualization of intact cells and their cytopathological characteristics. As stated in materials and methods, we consider a cell as hybrid when it has all CTC features together with CD45 expression. In addition, we also observed, in many cases, CD45 co-expressed with other epithelial (EpCAM) or fusion marker (MC1-R). Recently, Gast et al. used murine models to investigate cell fusion and demonstrated that leukocyte–cancer cell fusion produces hybrid cells that express the genetic and phenotypic characteristics of both maternal cells [49,50]. Other studies describe similar findings for macrophage markers, such as CD68, commonly expressed by tumor cells in histopathological sections [51,52,53,54]. Pawelek et al. proposed that the epithelial–mesenchymal transition in cancer could be the result of fusion between tumor and myeloid cells. Because macrophages are of mesenchymal origin, tumor-myeloid fusion could contribute to the aneuploidy and heterogeneity frequently observed in tumors. An inflammatory tumor microenvironment may also stimulate fusion between bone-marrow-derived cells and tumor cells. Fusion cells can also result from tumor cell phagocytosis by macrophages [21]. In the present study, it was noted that in some cells, CD45 expression occurred only on one side of the cell membrane, suggesting a fusion between two cells from different origins. Another hypothesis is that leukocytes send exosomes to primary tumors and to CTCs, which could explain why pieces of CD45-expressing membrane were observed in CTCs. Due to the important role of the immune system in the elimination of aberrant cells, escape from immune control is crucial for cancer growth and metastasis. Curiously, CD45 expression in CTCs was also found in cells that were automatically deleted by the CellSearch^®^ system, because cells expressing this marker are considered leukocytes regardless of their cytopathological features. Perhaps the sorting methods for CTCs need to be updated in order to include complex cells in disease analysis.

Although the present study used only a small sample size, it presents two major findings: the presence of hybrid cells in high grade ovarian cancer, as assessed by two different methods, and MC1-R as a prognostic marker for ovarian cancer. Additional studies, maybe multicenter, are needed to more thoroughly evaluate the function of MC1-R in ovarian cancer, as well as its role as fusion marker. Many important questions remain to be answered about the role of cell fusion in cancer with regard to its frequency, timing, causation and interaction with the immune system [18]. Therefore, cell fusion between tumor and immune cells offers a novel mechanism by which neoplastic cells gain phenotypic diversity, increasing opportunities for highly tuned subclones to overcome selection pressure and lead to tumor progression.

## 4. Materials and Methods

### 4.1. Patient Population and Study Design

This was a single-center, prospective, longitudinal study performed at the A.C. Camargo Cancer Center, São Paulo, Brazil, of patients with high-grade serous ovarian cancer monitored from November 2018 to May 2022. We included patients presenting platinum-sensitive recurrence, irrespective of *BRCA1* or *BRCA2* mutation. Patients were treated with standard of care treatment: a combination of platinum-based chemotherapy followed by PARP inhibitor maintenance (olaparib).

Three serial blood samples were collected. The first was collected at the time of patient enrollment (baseline), before starting treatment with PARPi (collection 1, CTC1). The second sample was taken 30 days after starting treatment with PARPi (first follow-up, CTC2), and the third sample was taken three months after starting treatment with PARPi (second follow-up, CTC3). Venous blood was collected from the antecubital vein in EDTA tubes, and the samples were stored at room temperature, with constant homogenization (in electric homogenizer) to prevent blood clotting, for a maximum of 6 h before being processed by the ISET system (Rarecells Diagnostics, Paris, France).

In addition, we included samples from locally advanced ovarian cancer with peritoneal carcinomatosis but no distant metastases, analyzed using the CellSearch system at the Department of Surgical Oncology, Institut du Cancer de Montpellier, ICM-Val d’Aurelle, France, and analyzed using CellSearch System (Menarini Silicon Biosystems, Huntingdon Valley, PA, USA), in parallel with the AC Camargo cohort.

### 4.2. Ethics

This project was approved by the ethics and research committee of A.C. Camargo Cancer Center, São Paulo, Brazil (approval number 2623/18). Written informed consent was obtained from all participants before their entrance into the study. In addition, patients that were recruited at the Institut du Cancer de Montpellier were recruited under the bioethics approval ‘CPP Sud Méditerranée III’ (reference number 2016.09.06).

### 4.3. Detection of CTCs and the Cell Fusion Phenotype

#### 4.3.1. ISET Assay

The ISET^®^ system (Isolation by SizE of Tumor cells, Rarecells, Paris, France) was used to isolate and analyze CTCs. Blood was drawn into EDTA tubes (BD Vacutainer^®^, BD, Belo Horizonte, MG, Brazil) with immediate gentle agitation. The sample was processed on the ISET platform according to manufacturer instructions. Briefly, 6 mL of whole blood were diluted up to 60 mL with a buffer containing 0.02% formaldehyde, incubated for 10 min at room temperature, and filtered through a membrane with 8 µm pore size. The filtration pressure was optimized to −10 kPa to preserve cell integrity. The membrane was then washed once with phosphate-buffered saline. After processing, filters were dried, wrapped in an aluminum sheet and stored at −20 °C until use.

#### 4.3.2. Immunocytochemistry

The module filtration had a membrane of 10 spots, making it possible to process blood samples of 10 mL. We analyzed 6 spots for each patient; all spots were analyzed for CD45 staining with another marker by dual color staining. After filtration, membranes were washed with PBS, disassembled from the filtration module, allowed to air-dry overnight and stored at −20 °C until staining. The spots membranes were submitted to dual color immunocytochemistry (ICC) (DAB+/Permanent Red; Dako^TM^, Santa Clara, CA, USA) on a 24-well plate. Antigen retrieval was then performed using Antigen Retrieval Solution (Dako^TM^). Cells were hydrated with tris-buffered saline (TBS) 1X for 20 min and permeabilized with TBS + Triton X-100 for 5 min, and endogenous peroxides were blocked with 3% hydrogen peroxide in the dark for 15 min. The spots were incubated with antibodies diluted in TBS 10% fetal calf serum. To amplify the antibody signal, the spots were incubated with Envision G/2 Doublestain System, Rabbit/Mouse (Dako^TM^) followed by 10 min of incubation with DAB+/Permanent Red (Dako^TM^). The spots were then washed with PBS between the steps. Cells were stained with hematoxylin for visualization of nuclei and cytoplasm and analyzed by light microscope (BX61-Olympus, Tokyo, Japan). To distinguish CTCs and cell fusion from white blood cells, we used anti-CD45 antibody. Circulating tumor cells (CTCs) were characterized based on the following cytopathological criteria: negative staining for CD45, nucleus size ≥ 12 µm, hyperchromatic and irregular nucleus, visible presence of cytoplasm and a high nucleus–cytoplasm ratio [9].

The classic definition of a CTC in cancer is a circulating cell that expresses a tumor antigen (usually EPCAM or cytokeratin) and does not express the CD45 pan-leukocyte antigen [55]. Leukocytes, including macrophages, normally express CD45. Therefore leukocyte/macrophage–CTC fusion cells must also express CD45 [56,57]. In our study, by using ISET, we identified the presence of CTCs based on the morphologic features observed through cytopathology rather than the expression of epithelial markers. It corroborates with studies developed by Patrizia Paterlini-Bréchot’s group, concerning kidney and prostate tumors, where they analyzed CTCs by the same method, by cytopathology characteristics, with 100% efficiency and 70% sensitivity. In addition, as far as we know, we are the first to demonstrate, together with CTCs, the identification and characterization of hybrid cells using the ISET method [35,36]. Thus, differently from the literature, in our study, CTC was considered to be a hybrid cell if it had the morphologic features of an epithelial cell and expressed CD45 alone or with MC1-R [24,25,31], or EpCAM [33]. The following antibodies were used: CD45 1:300 (Abcam: ab10559—Lot. GR3389366-1); MC1-R 1:700 (Abcam, Waltham, MA, USA): ab236734, lot GR3241231-10) and EpCAM 1:300 (Sigma, São Paulo, Brazil): HPA026761, lot D119365). Negative and positive controls were included in each ICC staining. For positive controls, we used cell lines A549 and MCF7 spiked in healthy blood, which, according to The Human Protein Atlas (http://www.proteinatlas.org/ (accessed on 10 September 2022)) express MC1-R and EpCAM, respectively. For negative controls we used cell lines SK-BR-3 and U-87-MG spiked in healthy blood, which, according to The Human Protein Atlas (http://www.proteinatlas.org/ (accessed on 10 September 2022)) do not express MC1-R and EpCAM, respectively. The cell lines were acquired from ATCC^®^ HTB-43™. For CD45, MC1-R and EpCAM expression analysis, cells were classified according to staining. No staining was considered negative, and any staining was classified as positive (Supplementary Figure S1).

#### 4.3.3. Chromogenic In Situ Hybridization

To confirm if the circulating CD45^+^/MC1-R^+^ or CD45^+^/EpCAM^+^ cells were hybrid cells, we performed qualitative detection of human chromosome 8 alpha satellites (CEN8) by chromogenic in situ hybridization. The presence of polyploidy was only assessed in the baseline collection from each patient. We used ZytoDot (REF: C-3016-400) stains on frozen ISET membranes. The membranes were hydrated with TBS 1X for 5 min at room temperature (RT) and then with 1% formaldehyde for 5 min at RT. Subsequent washes between steps were performed with distilled water. Membranes were incubated in hydrogen peroxide for 10 min at RT and in the dark. After washing with distilled water, membranes were incubated with cytology pepsin (ZytoVision, Bremerhaven, Germany) for 5 min at RT. Membranes were then washed in 70%, 90% and 100% ethanol for one minute each. After drying, the membranes were incubated with a CEN8 (10 µL) probe in the hybridizer at 75 °C (wet) for 5 min and then incubated overnight at 37 °C. After this step, washes were performed with wash buffer SSC for 5 min at RT. The membrane was incubated with wash buffer SSC at 80 °C for 5 min. After washing, the membranes were incubated with Anti-DIG/DNP-Mix (AB14) for 15 min at 37 °C (wet) in the hybridizer. After washing, HRP/AP-POLYMER-MIX (AB13) was applied, followed by AP-Red Solution (1–2 drops) at RT. The membrane was counterstained with 50% hematoxylin for 2 min at RT after washing. The slides were adhered with aqueous mounting medium and coverslipped (DAKO). The reading was performed under a bright field microscope. For CEN8 expression analysis, cells were classified according to the presence of expression. No expression was considered negative and any expression was classified as positive (Supplementary Figure S1). Notably, the spots analyzed by CISH were different from the spots analyzed by immunocytochemistry since the polycarbonate membrane would not resist the high temperatures used in CISH staining.

#### 4.3.4. The CellSearch^®^ System (Menarini Biosystems)

Blood samples were collected in CellSave tubes (Veridex, LLC, Huntingdon Valley, PA, USA) and processed within 96 h after blood collection. A total of 7.5 mL of blood was subjected to CTC enumeration using the CellSearch Epithelial Cell Kit. This procedure enriches the sample for cells expressing EpCAM using magnetic beads coated with anti-EpCAM antibodies and labels the cells with the dye fluorescent nucleic acid 4,2-diamidino-2-phenylindole dihydrochloride. Fluorescent monoclonal antibodies specific to leukocytes (CD45- alophycocyan) and epithelial cells (cytokeratin 8,18,19-phycoerythrin) are used to distinguish epithelial cells from leukocytes. The identification and enumeration of CTCs were performed using the CellSpotter Analyzer(Inc. DBA Sciotex, Newtown Square, PA, USA), a fluorescence microscopy system that allows for the digital reconstruction of cellular images. CTCs were defined as nucleated cells without CD45 and expressing cytokeratin. In our current study, we also selected EpCAM^+^CK^+^DAPI^+^CD45^+^ cells.

### 4.4. Statistical Analysis

A descriptive analysis (absolute data and frequency) was performed for all clinical-pathological variables. Continuous variables were categorized to evaluate the differences between the two groups: those who expressed in CTCs the markers MC1-R, CD45, EPCAM and CEN8 (hybrid cells) and those who did not. CTC count, cancer antigen 125 (CA125) levels, and age were dichotomized using the median value as a cut-off. χ^2^ or Fisher’s exact test was used to analyze categorical variables. Survival curves were plotted using the Kaplan–Meier method, and the difference between them was analyzed by the log rank test. RFS was defined as the time from baseline sample to disease recurrence or death by any cause. OS was defined as the time from baseline sample to death by any cause. All statistical analyses were performed using SPSS for Windows, version 15. The *p*-values were considered significant when ≤0.05.

## 5. Conclusions

In short, the present study indicated the association of polyploid/hybrid CTCs with RFS, especially for cells expressing MC1-R. More research is needed to elucidate the underlying mechanism, to better understand the biology of the metastatic cascade and how CTCs can escape the immune system. The main objective of future studies should be to determine the frequency of fusion cells in larger cohorts of human cancers in order to identify reliable markers and their role in malignant transformation and treatment resistance [58,59]. Although this study comprised only a small cohort, it opens new perspectives for the use of CTCs to monitor fusion events and treatment resistance in ovarian cancer. The study is preliminary and innovative, highlighting a new direction in the study of cancer and its interaction with cells of the immune system, in addition to the relationship between cell fusions and cancer progression.

## Figures and Tables

**Table 1:** Clinic-pathological variables in patients with high grade ovarian cancer.

Variables	No.	(%)
**Median age at baseline, years**	45 years (29–55)	
**FIGO stage**		
IIIc	8	57.1
IV	3	21.4
No information	3	21.4
**Treatment for platinum-sensitive recurrence**		
Carboplatin and Paclitaxel	14	100
**Treatment cycles**		
6 cycles	9	64.3
8 cycles	2	14.3
No information	3	21.4
**Recurrence site before the 1st collection**		
Pelvic	4	28.6
Pelvic and mediastinum	2	14.3
Mediastinum	1	7.1
Pelvic/retroperitoneum	1	7.1
Retroperitoneum	1	7.1
No information	5	35.7
**Surgery**		
Optimal surgery	8	57.1
Suboptimal surgery	3	21.4
No information	3	21.4
**Mutation**		
*BRCA1/BRCA2*	8	57.1
No mutation	3	21.4
No information	3	21.4
**CA125 pre olaparib treatment median**	268 (7–2183.72)	
Relapse post-olaparib treatment	6	42.9
**CEN 8**	9/11	81.8
**CTC/mL median (baseline)**	0.58 CTCs/mL (0.33–9.91)	
**CTC/mL median (1° follow-up)**	0.60 CTCs/mL (0.20–7.20)	
**CTC/mL median (2° follow-up)**	1 CTCs/mL (1.00–8.58)	

## Data Availability

Data supporting reported results can be found of https://dados.accamargo.org.br/redcap/?__messenger=open (last accessed on 20 September 2022).

## Supplementary Materials

The following supporting information can be downloaded at: https://www.mdpi.com/article/10.3390/ijms232314687/s1.
