# Supplementary material for: Fusion Cell Markers in Circulating Tumor Cells from Patients with High-Grade Ovarian Serous Carcinoma
Source: Int J Mol Sci. 2022 Nov 24;23(23):14687. doi: 10.3390/ijms232314687 (PMC9740150; doi:10.3390/ijms232314687)
Supplement: Supplementary file 1 [file ijms-23-14687-s001.zip › ijms-1982861-supplementary.pdf]

**Supplementary Materials**

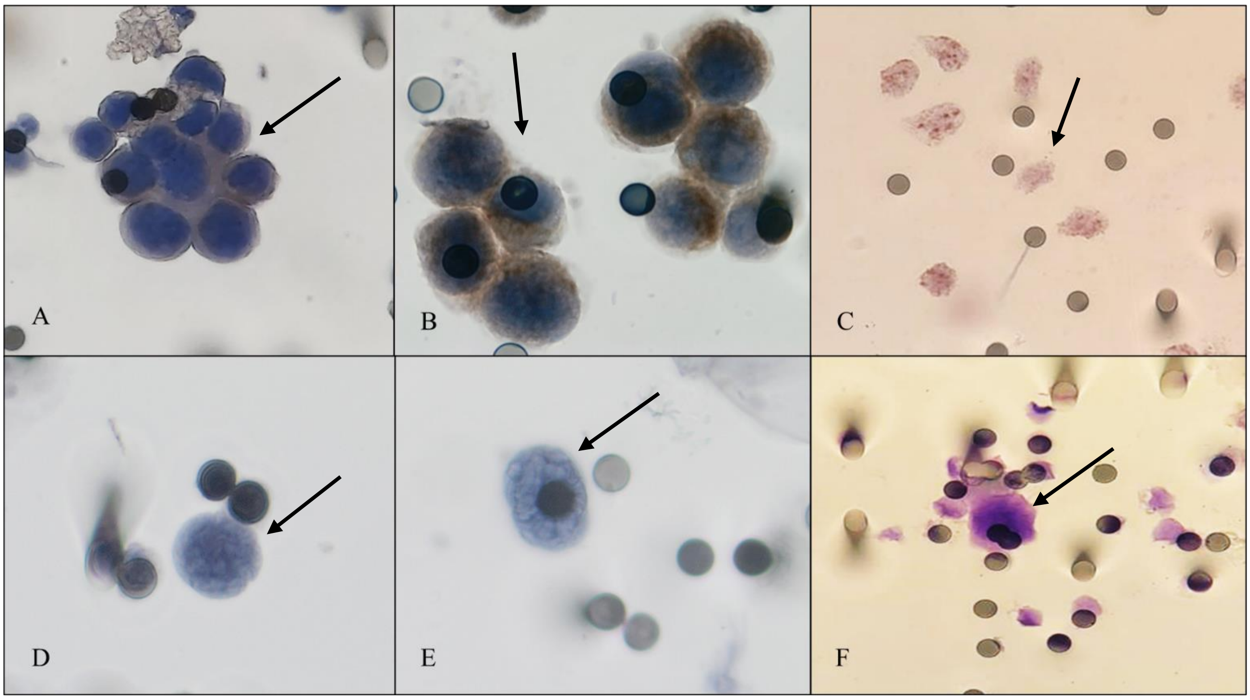


**Supplementary Figure S1. Pictures from healthy blood spiked with cell lines for antibodies control.** (**A**) Positive Control performed with healthy blood spiked with cell line A549 strain CCL-185 (ATCC ®), positive for MC1-R (visualized with DAB). (**B**) Positive Control. Healthy blood spiked with cell line MCF7 strain HTB-22 (ATCC ®), positive for EpCAM (visualized with DAB). (**C**) Positive Control. Healthy blood spiked with cell line A-549 strain CCL-185 (ATCC®), positive for CEN8. (**D**) Example of negative control counterstained with hematoxylin and isolated with healthy blood spiked with cell line SK-BR-3 HTB-30 strain, know to not express MC1-R protein. (**E**) Example of negative control. Healthy blood spiked with cell line U-87-MG HTB-14 strain, know to not express EpCAM protein. (**F**) Example of negative control. Healthy blood spiked with cell line SK-BR-3 HTB-30 strain, know to not express CEN8. The thick arrow represents the cells. Images were taken at ×200 magnification using a light microscope (Research System Microscope BX61—Olympus, Tokyo, Japan) coupled to a digital camera (SC100—Olympus, Tokyo, Japan)

**Suplementary Table S1.** Clinical data and protein expression in CTCs.

| | **Baseline (CTC1)** | | | | | **1º Follow-up (CTC2)** | | | **2º follow-up (CTC3)** | | | | | |
|---|---|---|---|---|---|---|---|---|---|---|---|---|---|---|
| Patients | **CTC/mL** | **CISH CEN8** | **MC1R** | **CD45** | **EpCAM** | **CTC/mL** | **MC1R** | **CD45** | **CTC/mL** | **MC1-R** | **CD45** | **EpCAM** | **Recurrence** | **CTM** |
| 1 | 4.66 | - | + | - | - | 0.75 | + | - | 0 | - | - | - | No | CTC1 |
| 2 | 0 | - | - | - | - | 1.33 | + | - | 0 | - | - | - | Yes | |
| 3 | 0.2 | + | - | - | - | 0 | - | - | 0 | - | - | - | Yes | |
| 4 | 1 | + | + | - | - | 0.66 | + | - | 0 | - | - | - | Yes | |
| 5 | 9.91 | + | + | - | - | 0.75 | + | + | 3.33 | + | - | - | Yes | |
| 6 | 1 | - | + | + | - | 0 | - | - | 0.33 | + | - | - | Yes | |
| 7 | 1.66 | + | + | - | - | 1.33 | + | + | 4.06 | + | + | - | Yes | |
| 8 | 0.2 | + | - | - | - | 3.66 | + | + | 8.58 | + | + | + | Yes** | CTC1, CTC2, CTC3 |
| 9 | 0.33 | + | + | + | - | 0 | - | + | 0 | - | - | - | No | |
| 10 | 0.5 | + | + | + | + | 7 | + | + | 1 | + | - | - | No | CTC3 |
| 11 | 0.66 | + | + | - | - | 0.33 | + | + | * | * | * | * | * | * |
| 12 | 1.08 | - | + | - | - | 0 | - | - | * | * | * | * | * | * |
| 13 | 0.66 | + | + | - | - | 0 | - | - | * | * | * | * | * | * |
| 14 | 0.2 | + | - | - | - | 0.33 | + | - | * | * | * | * | * | * |

Abbreviations: *: no blood collection; ** Last follow-up: 05.2022.
